# Characteristics and Functions of Infection-enhancing Antibodies to the N-terminal Domain of SARS-CoV-2

**DOI:** 10.20411/pai.v9i2.679

**Published:** 2024-06-18

**Authors:** Ruth I. Connor, Mrunal Sakharkar, C. Garrett Rappazzo, Chengzi I. Kaku, Nicholas C. Curtis, Seungmin Shin, Wendy F. Wieland-Alter, Jordan Wentworth, Daniel W. Mielcarz, Joshua A. Weiner, Margaret E. Ackerman, Laura M. Walker, Jiwon Lee, Peter F. Wright

**Affiliations:** 1 Department of Pediatrics, Geisel School of Medicine, Dartmouth Health, Lebanon, New Hampshire; 2 Department of Microbiology and Immunology, Geisel School of Medicine, Dartmouth College, Hanover, New Hampshire; 3 Adimab LLC, Lebanon, New Hampshire; 4 Thayer School of Engineering, Dartmouth College, Hanover, New Hampshire; 5 DartLab, Dartmouth Cancer Center, Geisel School of Medicine, Lebanon, New Hampshire

**Keywords:** SARS-CoV-2, COVID-19, antibodies, serum clonotypes, infection enhancement, FcR activation, variants of concern

## Abstract

**Background:**

Fcγ-receptor (FcγR)-independent enhancement of SARS-CoV-2 infection mediated by N-terminal domain (NTD)-binding monoclonal antibodies (mAbs) has been observed *in vitro*, but the functional significance of these antibodies *in vivo* is less clear.

**Methods:**

We characterized 1,213 SARS-CoV-2 spike (S)-binding mAbs derived from COVID-19 convalescent patients for binding specificity to the SARS-CoV-2 S protein, VH germ-line usage, and affinity maturation. Infection enhancement in a vesicular stomatitis virus (VSV)-SARS-CoV-2 S pseudovirus (PV) assay was characterized in respiratory and intestinal epithelial cell lines, and against SARS-CoV-2 variants of concern (VOC). Proteomic deconvolution of the serum antibody repertoire was used to determine functional attributes of secreted NTD-binding mAbs.

**Results:**

We identified 72/1213 (5.9%) mAbs that enhanced SARS-CoV-2 infection in a PV assay. The majority (68%) of these mAbs recognized the NTD, were identified in patients with mild and severe disease, and persisted for at least 5 months post-infection. Infection enhancement by NTD-binding mAbs was not observed in intestinal and respiratory epithelial cell lines and was diminished or lost against SARS-CoV-2 VOC. Proteomic deconvolution of the serum antibody repertoire from 2 of the convalescent patients identified, for the first time, NTD-binding, infection-enhancing mAbs among the circulating immunoglobulins directly isolated from serum. Functional analysis of these mAbs demonstrated robust activation of FcγRIIIa associated with antibody binding to recombinant S proteins.

**Conclusions:**

Functionally active NTD-specific mAbs arise frequently during natural infection and can last as major serum clonotypes during convalescence. These antibodies display functional attributes that include FcγR activation, and may be selected against by mutations in NTD associated with SARS-CoV-2 VOC.

**Figure:**
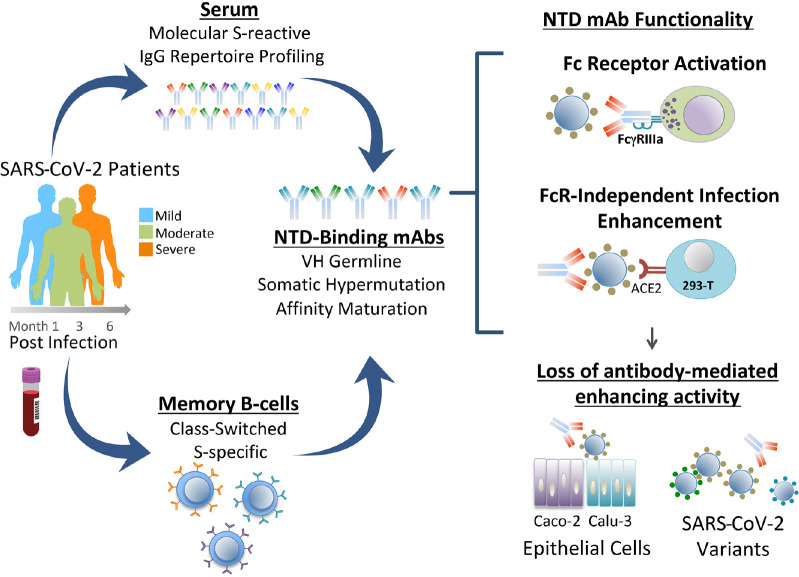


## INTRODUCTION

Identification of functional antibody responses to the severe acute respiratory syndrome coronoavirus-2 (SARS-CoV-2) spike (S) protein has contributed to an understanding of the immune correlates of protection and informed vaccine development. The S protein of SARS-CoV-2 projects from the virion surface as a trimer of heterodimers, each consisting of 2 subunits, S1 and S2, formed by furin cleavage. Within the S1 subunit, the receptor-binding domain (RBD) mediates binding of the virus to angiotensin-converting enzyme-2 (ACE2) on host cells and elicits neutralizing antibodies following natural infection and after vaccination [1–4]. The RBD is subject to mutation contributing to the emergence of SARS-CoV-2 variants with increased transmissibility and significantly reduced susceptibility to neutralization [5–7]. As the S protein constitutes the focus of many current vaccine platforms, considerable effort has been made toward understanding the evolution of the antibody repertoire and the functional impact of RBD mutations on neutralization of emerging SARS-CoV-2 variants of concern (VOC) [8].

Less well understood is the functional role and immunogenicity of the N-terminal domain (NTD) of the S1 subunit. The NTD has multiple glycosylation sites among 5 highly variable loop structures (N1-5) that serve to fine tune and facilitate virus entry into host cells [9–12]. NTD-binding antibodies can neutralize SARS-CoV-2, and these antibodies recognize epitopes within a convergent antigenic supersite [13–17], but also within epitopes outside this site [18]. Mutations in the NTD are present in all SARS-CoV-2 VOC to date and encompass residues constituting the neutralization supersite [13, 15] and those predicted to contribute to evasion of serum neutralizing antibodies [17], consistent with immune selection driving evolution of the virus in this subdomain [8].

A small number of NTD-binding antibodies have been found to enhance SARS-CoV-2 infection *in vitro* through Fcγ-receptor (FcγR)-independent mechanisms [19, 20]. These infection-enhancing antibodies recognize NTD epitopes outside the neutralization supersite and adjacent to the NTD variable loops [19, 20]. The significance of NTD-binding, infection-enhancing antibodies *in vivo* is less clear, as animal studies found minimal evidence of disease enhancement following antibody infusion and viral challenge in mouse and non-human primate models [19]. These results are supported by evidence that NTD-binding antibodies can have additional functional attributes including FcγR-mediated effector functions that may contribute to viral clearance and protection from disease [21]. However, detection of NTD-binding, infection-enhancing antibodies at a higher frequency in the sera of COVID-19 patients with severe disease has suggested a correlation between enhancing antibodies and disease severity [20]. Overall, these findings leave unanswered questions as to the significance and functional attributes of infection-enhancing antibodies directed to the NTD.

In the present study, we sought to determine the frequency, characteristics, and functional attributes of a panel of NTD-binding antibodies derived from a large pool of S-specific mAbs from 8 COVID-19 convalescent donors [22]. Our findings demonstrate a broad antibody response to the NTD with evidence of neutralizing and infection-enhancing activities associated with mAbs from donors with mild or severe COVID-19. We present evidence of loss of antibody functionality to Omicron variants that correlates with loss of binding to the variant S proteins. Moreover, we demonstrate for the first time the presence of NTD-binding, infection-enhancing antibodies as major clonotypes in the serum repertoire of 2 subjects and show the corresponding recombinant mAbs have FcγR-activating functionality, suggesting their potential to contribute to immune protection.

## METHODS

### Study Participants and Design

We recruited 8 participants with PCR-confirmed SARS-CoV-2 infection for this study from 8 April to 22 May 2020 [23]. Based on their infection dates, it is presumed the infecting strain was a Wuhan-Hu-1 (Wuhan) isolate. The clinical and biological characteristics of the participants have been reported in detail elsewhere [22–25]. In accordance with the study design, participants were enrolled within approximately 1 month (median 35.5 days) after SARS-CoV-2 diagnosis and were followed for at least 6 months. Two additional specimen collection visits occurred at approximately 2 months (Visit 2, median 95.5 days) and 5 months (Visit 3, median 153.5 days) after the initial visit. Based on clinical presentation and medical history, 5 patients were considered to have mild to moderate COVID-19 illness, and 3 had severe illness with hospitalization. Blood samples were processed to isolate B cells and serum in the Immune Monitoring Core Laboratory at the Dartmouth Geisel School of Medicine. The isolated B cells and serum were aliquoted and stored at −80°C until further use. The study was approved by the Dartmouth-Hitchcock Hospital (D-HH, Dartmouth Health, Lebanon, NH) Human Research Protection Program Institutional Review Board. All volunteers gave written informed consent using an approved D-HH template prior to participation in the study.

### Expression and Purification of IgGs and Fab Fragments

Monoclonal antibodies used for binding and functional assays were produced as full-length IgG1 proteins in *S. cerevisiae* cultures, as previously described [4]. Briefly, yeast cultures were incubated in 24-well plates at 30°C and 80% relative humidity with shaking at 650 RPM in Infors Multitron shakers. After 6 days of growth, the culture supernatants were harvested by centrifugation and IgGs were purified by protein A-affinity chromatography. IgGs bound to the agarose were eluted with 200mM acetic acid with 50mM NaCl (pH 3.5) and neutralized with 1/8 (v/v) 2 M HEPES (pH 8.0). Antigenic sites targeted by mAbs were evaluated using a panel of SARS-CoV-2 subunits and domains (RBD, NTD, Subunit 1 [S1], and a recombinant Subunit 2 [S2] that contains pre-fusion stabilizing mutations) as described in detail [22].

To generate Fab fragments, IgGs were digested with papain for 2 hours at 30°C followed by the addition of iodoacetamide to terminate the reaction. The mixtures were then passed over protein A agarose to remove Fc fragments and undigested IgG. The flow-through of the protein A resin was passed over either KappaSelect resin or LambdaFabSelect resin (Cytiva Life Sciences) for antibodies utilizing the kappa or lambda light chain classes. The Fabs captured on the resin surface were eluted using 200 mM acetic acid/50mM NaCl (pH 3.5) into 1/8th volume 2M Hepes (pH 8.0). Monovalent equilibrium dissociation constant (KD) affinities of Fabs were measured using biotinylated antigens (100nM) immobilized on streptavidin biosensors (1.0-2.0 nm) (Molecular Devices) as described [22].

### Isolation and Characterization of Serum mAbs

Methods for the isolation and characterization of serum antibodies to SARS-CoV-2 from these patients are described in detail in a companion paper [26]. In brief, serum IgG was enriched by passing serum through Protein G agarose columns (Thermo Fisher, 20397) and eluting the bound antibodies with 100mM glycine-HCl, pH 2.7. Purified IgG was digested into F(ab')2 with 25 μg of IdeS protease per 1 mg of IgG and then incubated with Strep-Tactin agarose (IBA-Lifesciences, 2-1206-025) for 1 hour to remove the IdeS protease. For each sample, F(ab')2 were further enriched by passage through affinity columns of N-hydroxysuccinimide (NHS)–activated agarose resin coupled with recombinant Wuhan SARS-CoV-2 S or RBD proteins as described [26]. Samples were then denatured/alkalized, followed by digestion with trypsin (1:30 (w/w) trypsin/protein) for 16 hours at 37°C. The resulting peptides were purified using a Hypersep SpinTip C-18 (Thermo Fisher Scientific) and analyzed by liquid chromatography-tandem mass spectrometry (LC-MS/MS). Sequence data for V_H_ and V_L_ from each donor was used to construct protein sequence databases. The V_H_ sequences were then grouped into clonotypes based on hierarchical clustering. Selection of antibody sequences for recombinant expression was based on the combination of V_H_:V_L_-paired databases and proteomics data. These genes were then purchased as eBlocks (Integrated DNA Technologies) and cloned into the pcDNA3.4 vector (Invitrogen). Heavy and light chain plasmids for each mAb were transfected into Expi293 cells, and the expressed antibodies purified from cell culture supernatants using Protein G agarose. Using a matched donor-specific database of putative B-cell receptor sequences at Visit 1, the secreted NTD-binding mAbs were matched to mAbs cloned from the memory B-cell population of each participant [26]. Recombinant antibodies representing each of the 12 most abundant serum IgG clonotypes from 2 donors with moderate and severe symptoms (participants 3546 and 3548, respectively) were characterized by ELISA and biolayer interferometry (BLI) for binding specificity, and for neutralization and Fc-mediated functional activities as described [26].

### VSV-SARS-CoV-2 Pseudovirus Neutralization Assay

Neutralization assays were performed using a VSV-SARS-CoV-2 pseudovirus system as previously described [22–25, 27]. Plasmids expressing the SARS-CoV-2 S protein were obtained from Addgene: pcDNA3.3-SARS2-B.1.617.2 (Delta) was a gift from David Nemazee (Addgene plasmid #172320; http://n2t.net/addgene:172320; RRID:Addgene_172320) [28]; pTwist-SARS-CoV-2Δ18 B.1.1.529 (Omicron) was a gift from Alejandro Balazs (Addgene plasmid #179907; http://n2t.net/addgene: 179907; RRID:Addgene_179907) [29]; pcDNA3.3_SARS2_omicron BA.1 (Addgene plasmid #180375; http://n2t.net/addgene: 180375; RRID:Addgene_180375) and pcDNA3.3_ SARS2_omicron BA.2 (Addgene plasmid #183700; http://n2t.net/addgene: 180700; RRID:Addgene_180700) were gifts from David Nemazee [30] and were used to create VSV-SARS-CoV-2 S pseudoviruses [27]. The pcDNA3.3 SARS-CoV-2-Beta S expression plasmid was created inhouse by site-directed mutagenesis of pcDNA3.3 SARS-CoV-2 S to incorporate mutations (L18F, D80A, D215G, Δ242-244, R246I, K417N, E484K, N501Y/T, D614G and A701V) associated with the Beta variant S. To measure neutralization, individual mAbs were diluted to 50nM (7.5 μg/ml) and incubated with VSV-SARS-CoV-2 S pseudoviruses for 1 hour at 37°C before adding to either 293T-hsACE2 (Integral Molecular), Caco-2 (ATCC HTB-37), or Calu-3 (ATCC HTB-55) cells. Control wells contained pseudovirus with media and no added antibody. The cells were incubated at 37°C, 5% CO_2_ for 24 hours, after which luciferase activity was measured in cell lysates using the Bright-Glo system (Promega, Madison, WI) with a Bio-Tek II plate reader. Luciferase activity was expressed in relative light units, RLU), and the percentage neutralization was calculated as 100-(mean RLU test wells/mean RLU positive control wells) x 100.

### Measurement of ACE2 Expression

Flow cytometery was used to assess ACE2 expression on the cell surface of 293T-ACE2, Caco-2, and Calu-3 cells. The cells were first removed from culture flasks by gentle scraping, centrifuged, and resuspended in PBS for flow cytometry staining. Cells were stained for viability using LiveDead Blue Fixable Dye (Invitrogen) for 30 minutes at room temperature, washed and resuspended in 1.25 mg/mL unlabeled human IgG to block Fc receptor binding. Cells were then stained with either anti-ACE2-Alexa Fluor® 647-conjugated antibody (R&D Systems) or an isotope-matched control mouse IgG2A antibody (R&D systems) for 30 minutes at room temperature. Free antibody was removed via centrifugation and cells were resuspended in 0.5% paraformaldehyde. Cells were then acquired on a ZE-5 cytometer (Bio-Rad) and ACE2 expression was analyzed using FlowJo software (BD Biosciences). Data is presented as mean fluorescent intensity (MFI) for the ACE2 and isotype control antibodies.

### Monoclonal Antibody Binding ELISA

For antibody binding studies, 96-well ELISA plates were coated with 50 μL per well of recombinant SARS-CoV-2 (Wuhan) NTD, RBD, S2 proteins, or recombinant purified SARS-CoV-2 S prefusion trimeric proteins from Wuhan, Beta, Delta, Omicron BA.1, or Omicron BA.2 (Immune Technology Corp.) diluted to 5 mg/mL in 1X PBS and incubated overnight at 4°C. Wells were washed 3 times with PBS and blocked with 5% non-fat dry milk (NFDM)-PBS for 1 hour at 37°C. After removal of blocking buffer, test mAbs diluted to 100nM in 5% NFDM-PBS were added and incubated for 1 hour at 37°C. Plates were then washed 3 times with PBS and secondary cross-adsorbed anti-human Fab-HRP (Jackson ImmunoResearch Laboratories) detection antibody was added at 1:10000 dilution in 5% NFDM-PBS for 1 hour at 37°C. After washing 3 times with PBS, 50 μL per well of the 1-Step™ Ultra TMB-ELISA Substrate Solution (Thermo Fisher Scientific) was added to detect binding followed by addition of an equal volume of the stop reagent as per manufacturer recommendations. Absorbance was measured at 450 nm wavelength using a Bio-Tek II plate reader.

### FcγRIIIa Activation Reporter Assay

The ADCC potential of individual monoclonal antibodies was measured as described [24] using a Jurkat Lucia NFAT cell line (InvivoGen, jktl-nfat-cd16) cultured according to the manufacturer's recommendations, in which engagement of FcγRIIIa (CD16) on the cell surface leads to the secretion of luciferase. One day prior to running the assay, a high-binding 96-well plate was coated with 1 µg/mL SARS-CoV-2 S proteins from Wuhan, Beta, Delta, Omicron BA.1, or Omicron BA.2 (Immune Technology) at 4°C overnight. Plates were then washed with PBS + 0.1% Tween20 and blocked at room temperature for 1 hour with PBS + 2.5% BSA. After washing, monoclonal antibodies were diluted to a final concentration of 5 µg/mL and added to plates with 100,000 cells/well in growth medium lacking antibiotics in a final volume of 200 µL per well. The plates were cultured at 37°C, 5% CO_2_ for 24 hours. The following day, 25 µL of supernatant was drawn from each well and transferred to an opaque, white 96 well plate, to which 50 µL of QUANTI-Luc substrate was added and luminescence immediately read on a Bio-Tek II plate reader. For negative control wells we substituted assay medium for sample, while 1x cell stimulation cocktail (Thermo Fisher Scientific) plus an additional 2 μg/mL ionomycin were used to induce expression of the transgene as a positive control.

**Statistics.** Data were analyzed by linear regression to determine the coefficient of determination (R^2^) as indicated in the figure legends. Data values are presented as mean±SEM. Student's *t* test (2-tailed) was used to determine significance between groups. A *P* value of less than 0.05 was considered significant.

## RESULTS

### Frequency and Binding Specificity of SARS-CoV-2 Infection-enhancing mAbs

The frequency of FcγR-independent, infection-enhancing antibodies to SARS-CoV-2 was determined by measuring the neutralizing or infection-enhancing activities of 1,213 mAbs derived from a previous longitudinal study of B-cell evolution in 8 COVID-19 convalescent donors [22]. Antibodies were cloned from S-specific, class-switched memory B-cells at 3 visits with median times of 35.5 (Visit 1), 95.5 (Visit 2) and 153.5 (Visit 3) days post-infection. To evaluate functional activity, mAbs were screened at 50 nM (7.5 μg/mL) in a pseudovirus (PV) reporter assay using vesicular stomatitis virus bearing the Wuhan SARS-CoV-2 S (VSV SARS-CoV-2 S). Neutralization was defined as a ≥80% decrease in PV infection relative to virus-only controls, while enhancement was defined as a ≥50% increase in PV infection compared to virus-only controls.

Based on these criteria, neutralizing antibodies comprised 13.0% (158/1213) of the total number of antibodies screened and were found with increasing frequency across Visits 1, 2, and 3 (5.6%, 13.2%, 16.9%, respectively) (Table 1). By comparison, infection-enhancing antibodies were found less often, but still comprised 5.9% (72/1213) of all antibodies screened and increased in frequency from Visit 1 (2.6%) to Visit 3 (7.7%). These findings suggest that SARS-CoV-2 infection-enhancing antibodies are present throughout the COVID-19 convalescent period and persist for at least 5 months post-infection.

**Table 1.** Frequency of SARS-CoV-2 Neutralizing and Infection-enhancing mAbs

SARS-CoV-2 Spike (S)-binding mAbs	VISIT 1	VISIT 2	VISIT 3	TOTAL
Screened (N)	305	334	574	1213
Neutralizing ≥80% (N) (%)	17 (5.6)	44 (13.2)	97 (16.9)	158 (13.0)
Enhancing ≥50% (N) (%)	8 (2.6)	20 (6.0)	44 (7.7)	72 (5.9)

mAbs were cloned at a median of 35.5 (Visit 1), 95.5 (Visit 2) and 153.5 (Visit 3) days post-infection from 8 COVID-19 convalescent patients.

**Figure 1.**
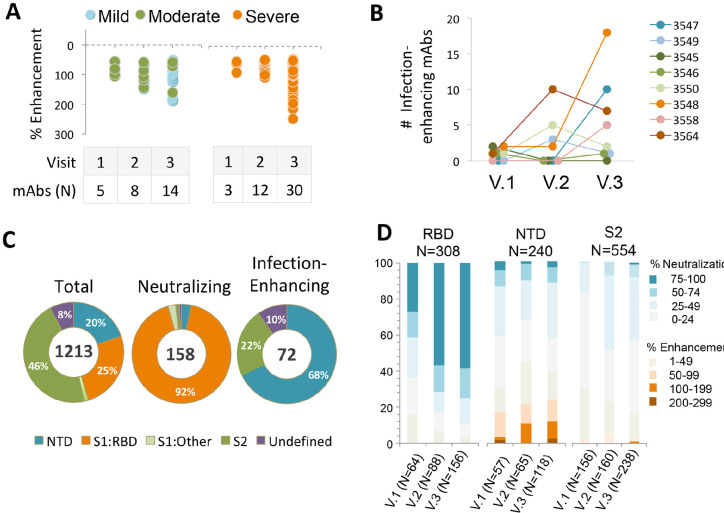
**Frequency and binding specificity of SARS-CoV-2 infection-enhancing mAbs.** (A) Infection was measured in a VSV-SARS-CoV-2 S (Wuhan) pseudovirus (PV) reporter assay in the presence of monoclonal antibodies (mAbs) from 8 COVID-19 convalescent patients with mild (N=2), moderate (N=3), or severe (N=3) illness. Median days post-infection were 35.5 (Visit 1), 95.5 (Visit 2), and 153.5 (Visit 3). Infection enhancement was defined as an increase in infection ≥50% over virus-only controls. (B) Number of infection-enhancing mAbs identified for individual COVID-19 donors with mild (3547, 3549), moderate (3545, 3546, 3550), or severe (3548, 3558, 3564) illness in longitudinal sampling from Visits 1-3. (C) Binding specificity of total, neutralizing, and infection-enhancing mAbs to recombinant SARS-CoV-2 (Wuhan) NTD, S1:RBD, S1:Other (non-RBD), or S2 proteins. The mAbs were tested from all participants across all visits. (D) Proportion of infection-enhancing or neutralizing activities among mAbs binding to recombinant SARS-CoV-2 Wuhan RBD, NTD or S2. Data represent mAbs from all participants across visits 1-3.

Further analysis of antibody distribution among donors with different COVID-19 outcomes revealed the presence of infection-enhancing antibodies in individuals with mild, moderate, or severe illness (Figure 1A), suggesting these antibodies are not restricted to those with severe disease and may persist in individuals with a mild course of illness. Infection-enhancing mAbs were identified in all donors at one or more visits (Figure 1B), with higher cumulative numbers across all visits in patients with severe disease (45/642; 7.0%) as compared to those with mild-to-moderate illness (27/571; 4.7%). However, given the small number of patients studied, this difference failed to reach statistical significance, and it was not possible to determine whether this difference is biologically meaningful.

We next determined the binding specificity of neutralizing and infection-enhancing antibodies to the RBD, NTD, and S2 domains. As previously reported [22], the vast majority (92%) of neutralizing mAbs recognized the RBD with a small number binding to regions outside the RBD (Figure 1C). In comparison, most infection-enhancing antibodies were directed to the NTD (68%) with a smaller percentage (22%) recognizing S2. These results are consistent with prior reports describing several FcγR-independent, infection-enhancing mAbs, all of which bound epitopes within the NTD [19, 20, 31], and further suggest that additional binding specificities associated with infection enhancement may encompass regions of S2.

Further analysis of the functional distribution of neutralizing and infection-enhancing antibodies confirmed that the majority of neutralizing activity was associated with mAbs directed to the RBD, while the NTD was targeted by both infection-enhancing antibodies and a small number of neutralizing mAbs (Figure 1D). The S2 domain appeared to be comparatively functionally neutral with neither potent neutralizing nor infection-enhancing mAbs binding to this region, suggesting these antibodies recognize post-fusion epitopes on S2 that are not involved in modulation of virus infectivity.

### Unique VH Germline Characteristics of NTD-binding and Infection-enhancing Antibodies

**Figure 2.**
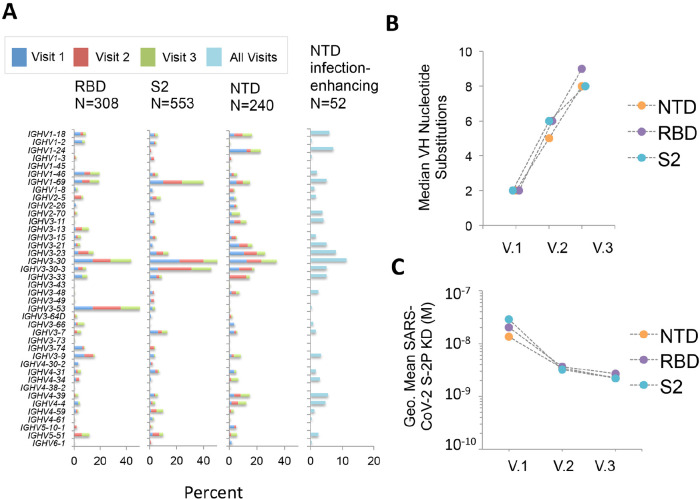
**Characteristics of NTD-binding, infection-enhancing mAbs.** (A) Frequency of VH germline usage at Visits 1-3 for mAbs binding to SARS-CoV-2 RBD, NTD, and S2, and for NTD-binding mAbs with infection-enhancing activity across all visits. (B) Median number of VH nucleotide substitutions for NTD-, RBD-, or S2-specific mAbs from all donors at Visits 1-3. (C) Geometric mean Fab binding affinities (equilibrium dissociation constant, K_D_) to recombinant, proline-stabilized S (SARS-CoV-2 S-2P) for mAbs from all donors at Visits 1-3. The mAbs that did not display detectable Fab binding are excluded from this analysis.

We next determined characteristics of the NTD-binding mAbs by analyzing the variable region of the antibody heavy chain (VH). The NTD-binding mAbs were first compared to antibodies directed to the RBD and S2 domains (Figure 2). In agreement with our prior results [22], we found increased representation of mAbs utilizing VH3-30 and VH3-53 germline gene segments among RBD-binding antibodies (Figure 2A). Similarly, S2-binding mAbs utilized a more focused set of VH germline segments including VH1-69, VH3-30 and VH3-30-3. In contrast, NTD-binding mAbs used a diverse array of VH genes, and this broad representation was mirrored in the infection-enhancing antibodies, which utilized VH germline segments including VH1-18, VH1-24, VH1-69, VH2-70, VH3-11, VH3-30, VH3-30-3, and VH4-39. Across all donors, the level of VH somatic hypermutation among NTD-binding mAbs increased from Visit 1 to Visit 3 (Figure 2B), with a median number of VH nucleotide substitutions of 2, 6, and 8 for sequential visits. NTD-binding mAbs also displayed increases in binding affinity to a proline stabilized, recombinant SARS-CoV-2 S protein (S2-P) across Visits 1-3 (Figure 2C) consistent with antigen-driven maturation of antibodies to this region.

### Infection-enhancing Activity of NTD-binding mAbs to SARS-CoV-2 Variants

**Figure 3.**
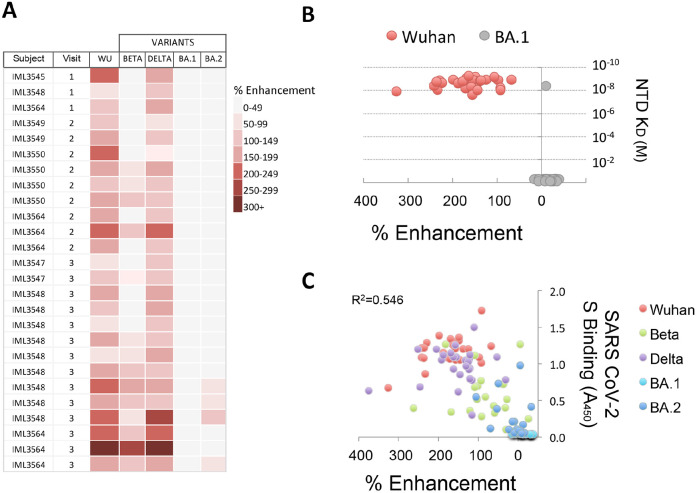
**Infection-enhancing activity of NTD-binding mAbs to SARS-CoV-2 variants.** (A) NTD-binding mAbs representing clones from multiple donors at Visits 1-3 were tested for infection-enhancing activity in a VSV-SARS-CoV-2 S pseudovirus (PV) assay. Infection-enhancement was measured using PV bearing the S from Wuhan (WU), Beta, Delta, or Omicron (BA.1, BA.2). (B) Percentage infection enhancement by NTD-binding mAbs to Wuhan- and BA.1-PVs and binding affinities (equilibrium dissociation constant, K_D_) of Fab from each mAb to recombinant Wuhan and BA.1 NTD proteins. (C) Correlation of percentage infection-enhancement and ELISA binding activity (Absorbance 450 nm) to recombinant S proteins from Wuhan and SARS-CoV-2 variants (Beta, Delta, Omicron BA.1, Omicron BA.2) for NTD-binding mAbs. The coefficient of determination (R^2^) was calculated by linear regression.

We next determined whether the infection-enhancing activity of NTD-binding mAbs also occurred with SARS-CoV-2 VOC. Individual VSV-based pseudoviruses (PVs) were created bearing SARS-CoV-2 S from Beta, Delta, or Omicron (BA.1, BA.2) variants. NTD-binding, infection-enhancing mAbs (N=26) representing clones from multiple donors across Visits 1-3 were tested to determine changes in activity to each variant PV as compared to a Wuhan-PV. The majority of mAbs (24/26) enhanced infection of the Delta-PV at levels comparable to those of the Wuhan-PV, while approximately half (14/26) retained enhancing activity to the Beta-PV (Figure 3A). However, none of the mAbs displayed infection-enhancing activity with the Omicron BA.1-PV, and only a small number (4/26) enhanced infection of the Omicron BA.2-PV. The absence of infection-enhancing activity to the Omicron BA.1-PV was associated with near-complete loss of antibody binding to recombinant BA.1 NTD protein (Figure 3B), suggesting that mutations in the NTD of BA.1 abrogate the functional epitopes recognized by these mAbs. Overall, the level of enhancement of PV infection was correlated with the level of antibody binding to the respective variant S proteins by ELISA (R^2^=0.546) (Figure 3C), confirming the association of antibody binding with functionality.

### Mechanism of Infection-enhancement of SARS-CoV-2

**Figure 4.**
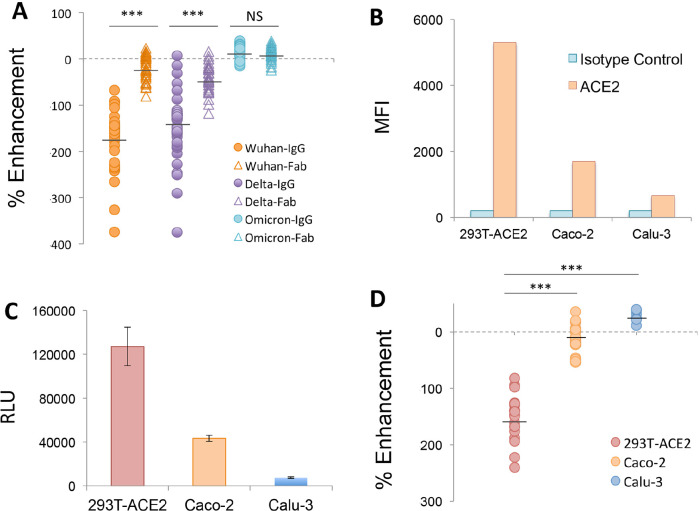
**Mechanism of infection-enhancement of SARS-CoV-2.** (A) Infection-enhancing activity of IgG and purified Fab generated from a panel of NTD-binding, infection-enhancing mAbs. Enhancement was measured in VSV-SARS-CoV-2 S pseudovirus (PV) assays using PV bearing S from Wuhan, Delta, or Omicron variants. Mean values for percentage enhancement by IgG and Fab against each PV were compared using a 2-tailed Student's *t* test, ***P<0.0001, NS=not significant. (B) Flow cytometry of cell-surface ACE2 expression on 293T-ACE2, intestinal (Caco-2), and respiratory (Calu-3) epithelial cell lines as compared to an isotype matched IgG2A control antibody. MFI=Mean Fluorescent Intensity. (C) Infection of 293T-ACE2, Caco-2 and Calu-3 epithelial cell lines with a Wuhan-PV. Luciferase activity was measured in cell lysates 24 hours after infection and expressed as the median of relative light units (RLU) for 5 replicate wells. (D) Percentage enhancement of Wuhan-PV infection by NTD-binding mAbs in 293T-ACE2, Calu-3, and Caco-2 cells. Mean values for percentage enhancement of infection between 2 cell types were compared using a 2-tailed Student's *t* test, ***P<0.0001.

A proposed mechanism for FcγR-independent enhancement of SARS-CoV-2 infection involves bivalent binding of antibodies to the NTD of adjacent S1 subunits, triggering the open conformation of the RBD and increased binding to ACE2 [20]. We further probed this mechanism by comparing the enhancing activity of IgG and purified Fab generated from a panel of NTD-binding, infection-enhancing mAbs (Figure 4). Our results demonstrate enhancement of Wuhan-PV infection in the presence of IgG mAbs that was lost when equimolar concentrations of Fab for each mAb were used instead of IgGs, confirming that bivalent antibody binding is required for infection-enhancing activity *in vitro* (Figure 4A). Similar results were found comparing IgG and Fab activities with a Delta-PV, whereas no enhancement was seen with an Omicron BA.1-PV using either IgG or Fab. This latter result is consistent with the observed loss of antibody binding to the BA.1 NTD protein (Figure 3B).

Our results also confirm that antibody-mediated enhancement of SARS-CoV-2 in these assays is independent of engagement with FcR. First, the 293T-ACE2 target cells used for infection do not express FcR and, further, all antibodies used in these experiments were produced in a yeast expression system and are aglycosylated, thus precluding most antibody interaction with FcR. Taken together, these results support a model of bivalent antibody binding to NTD as a necessary component for FcR-independent enhancement of SARS-CoV-2 *in vitro*.

As increased binding to ACE2 is also integral to this proposed mechanism [20], we next evaluated the impact of different ACE2-expressing target cells on the infection-enhancing activity of NTD-binding mAbs. Prior reports of infection-enhancing mAbs [20], as well as our own assays [22–24], utilize 293T cells that highly express ACE2 as targets for SARS-CoV-2 PV infection. In comparison, biologically relevant target cells for SARS-CoV-2 include epithelial cells lining the respiratory and gastrointestinal tracts, which may express lower levels of ACE2 [32–35]. We found respiratory (Calu-3) and intestinal (Caco-2) epithelial cell lines expressed significantly lower levels of cell surface ACE2 (Figure 4B) and had lower levels of PV infection after 24 hours (Figure 4C) as compared to 293T-ACE2 cells. Moreover, when compared with virus-only controls in each cell type, we found no evidence of infection enhancement by NTD-binding mAbs in either Calu-3 or Caco-2 cells (Figure 4D). This finding suggests that antibody-mediated enhancement of SARS-CoV-2 may be conditional upon the choice of target cells and may be less likely to occur in epithelial cells expressing lower ACE2 in the respiratory or gastrointestinal tracts.

### Expanded Functional Activity of NTD-binding Serum mAbs

**Figure 5.**
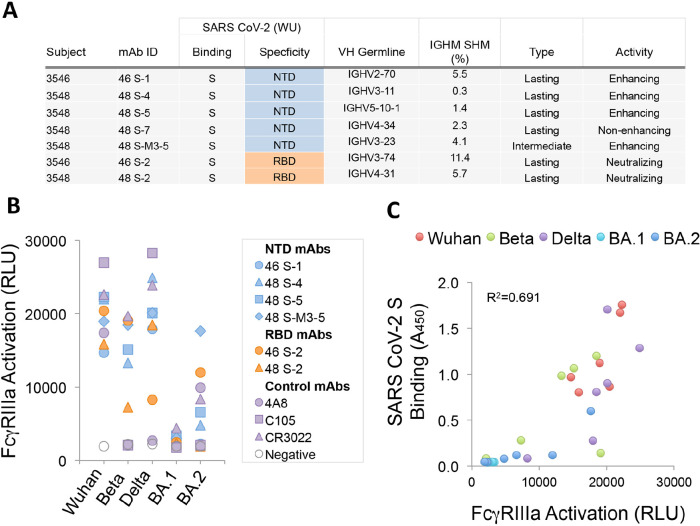
**Features and functional activity of NTD-binding, infection-enhancing serum mAbs.** (A) IgG mAbs representing major serum clonotypes from 2 donors (3546, 3548) were characterized for SARS-CoV-2 binding specificity, variable heavy chain (VH) germline use, percentage immunoglobulin heavy chain somatic hypermutation (IGHM SHM), and categorized as lasting (detected at Visit 3), intermediate (detected at Visit 2, but not Visit 3), or transient (detected only at Visit 1) as described (30). Functional activity was determined using a VSV-SARS-CoV-2 S (Wuhan) pseudovirus (PV) assay to measure neutralization or infection-enhancement as reported (30). (B) Serum mAbs binding NTD (46 S-1, 48 S-4, 48 S-5, 48 S-M3-5), RBD (46 S-2, 48 S-2), or control mAbs (4A8, C105, CR3022) were tested for activation of FcγRIIIa in a luciferase reporter-cell assay with recombinant S from Wuhan, Beta, Delta, or Omicron (BA.1, BA.2). FcγRIIIa activation was measured as relative light units (RLU). (C) Correlation of FcγRIIIa activation (RLU) and SARS-CoV-2 S binding by ELISA (Absorbance 450 nm) for NTD (46 S-1, 48 S-4, 48 S-5, 48 S-M3-5) and RBD (46 S-2, 48 S-2) binding mAbs to pre-fusion stabilized trimeric S from Wuhan, Beta, Delta or Omicron (BA.1, BA.2) variants. The coefficient of determination (R^2^) was calculated by linear regression.

The significance of antibody-mediated enhancement to the pathogenesis of SARS-CoV-2 has been debated following results in animal models, which found no evidence of enhanced disease in most animals infused with an NTD-binding, infection-enhancing mAb prior to virus challenge [19]. However, other studies in humans have demonstrated increased frequency of these antibodies in the sera of COVID-19 patients with severe disease [20]. We sought to examine the functionality of serum antibodies from 2 of the 8 COVID-19 convalescent patients in our cohort. These 2 patients (3546, 3548) had moderate and severe illness, respectively, and were evaluated within the context of a study of their serum antibody repertoires to SARS-CoV-2 [26]. A total of 12 unique mAbs, representing the most abundant serum clonotypes in each patient, were recombinantly expressed in Expi293 cells [26]. These antibodies are fully glycosylated and capable of engagement with FcγR. Five of these mAbs were found to bind the NTD, of which 4 enhanced infection with SARS-CoV-2 PVs *in vitro* [26] (Figure 5A).

The 4 NTD-binding, infection-enhancing mAbs were evaluated for additional functionality by measuring their ability to activate FcγRIIIa expressed on reporter cells as a surrogate measure of antibody-dependent cellular cytotoxicity (ADCC). Control antibodies included 4A8, an NTD-specific, neutralizing mAb [14], RBD-specific, neutralizing mAbs from the same participants (46 S-2, 48 S-2), and 2 additional RBD-specific neutralizing mAbs, C105 and CR3022 [36–38]. In assays using recombinant S proteins as antigens, all 4 serum-derived NTD-binding mAbs activated FcγRIIIa at levels comparable to serum-derived RBD-binding mAbs and to the positive control antibodies (Figure 5B). Evaluation of additional S proteins from Beta, Delta, and Omicron BA.1 and BA.2 variants revealed diminished activation of FcγRIIIa by some mAbs to Beta-S and complete loss of FcγRIIIa activation to BA.1-S by all mAbs. FcγRIIIa activation was correlated with the level of antibody binding to the recombinant S proteins from each variant by ELISA (R^2^=0.691) (Figure 5C), suggesting that mutations in epitopes recognized by these mAbs are responsible for the observed loss of functionality. These results are consistent with a prior study of FcR-mediated effector function by an NTD-binding mAb [21] and further suggest that NTD-binding antibodies that display infection-enhancing properties *in vitro* also have the capacity to activate FcγRIIIa and may therefore have the potential to mediate ADCC. These results also align with data demonstrating additional FcγR-mediated effector functions associated with these same serum-derived mAbs, including antibody-dependent cellular phagocytosis (ADCP) and antibody-dependent complement deposition (ADCD) [26].

## DISCUSSION

Characterization of the functional antibody responses to the SARS-CoV-2 S protein is important for understanding the immune correlates of protection and for informing vaccine development. Most current vaccine platforms focus on induction of neutralizing antibodies to the RBD of the S1 subunit, while less attention has been paid to the characteristics and functional attributes of antibodies to the S1 NTD. Both neutralizing [13–18] and infection-enhancing antibodies [19, 20, 31] directed to the NTD have been described, but their role, if any, in modulating SARS-CoV-2 infection and disease is less clear. In the present study, we examined antibody responses to the NTD using mAbs derived from longitudinal sampling of B cells collected from 8 COVID-19 convalescent patients. Our results reveal several novel aspects of the antibody response to the NTD and the functionality of NTD-binding mAbs.

First, by screening a large collection of S-specific mAbs derived from the memory B-cell populations of donors with mild, moderate, or severe COVID-19, we found infection-enhancing mAbs present in all donors irrespective of disease severity, and these antibodies persisted for up to 5 months post-infection. Infection-enhancing mAbs represented 5.9% of the total number of antibodies screened—roughly half the number of neutralizing mAbs identified—suggesting they may constitute a more significant pool of antibodies than previously recognized. Moreover, as compared to the RBD and S2 domains, we found broader representation of VH germline segments among antibodies binding the NTD, suggesting the possibility of greater diversity among NTD-binding specificities. Functional analysis further supported this observation by revealing both neutralizing and infection-enhancing activities associated with NTD-binding mAbs as compared to RBD-binding mAbs, which represented the majority of neutralizing activity. Overall, these findings suggest the NTD elicits antibodies with the potential for diverse functionality *in vivo*.

Next, we confirmed and extended results on the proposed mechanism of FcγR-independent enhancement of SARS-CoV-2 infection [20]. Our findings support observations that infection enhancement is mediated predominantly by NTD-binding mAbs and is predicated on bivalent antibody binding to S. Antibody binding is modeled to trigger the open conformation of RBD and to engage multiple ACE2 receptors on the host cell surface [20]. Earlier studies demonstrated enhancement of SARS-CoV-2 infection by NTD-binding mAbs in high-ACE2 expressing 293T cells and Huh7 cells, a human liver carcinoma cell line that expresses ACE2 [20, 39]. However, when using epithelial cell lines derived from human lung (Calu-3) and intestine (Caco-2), we found no evidence of SARS-CoV-2 infection enhancement by NTD-binding mAbs. Both Caco-2 and Calu-3 cells express ACE2 and are widely used to characterize SARS-CoV-2 infection *in vitro* [40–44]. The relative absence of infection enhancement with NTD-binding antibodies in these cells may result from lower ACE2 expression, a possibility that aligns with the observed impact of ACE2 expression levels on experimental measures of antibody neutralization of SARS-CoV-2 [45]. When considered alongside the functional requirement of bivalent antibody binding to NTD, it is possible that limiting levels of ACE2 on target cells may preclude active engagement of multiple receptors, thereby abrogating any enhancing effect of these antibodies on virus infectivity. While we cannot rule out post-entry effects on PV infection in these cells, our findings suggest the choice of target cell plays an important role in the phenotypic expression of FcγR-independent antibody enhancement of SARS-CoV-2 and raise the possibility that enhancement may be less likely to occur in epithelial cells in the respiratory or gastrointestinal tracts. Overall, it remains unclear whether this mechanism is widely, or even functionally, operative *in vivo*, particularly in light of results from animal studies, which found limited evidence of SARS-CoV-2 antibody-mediated disease enhancement [19].

**Figure 6.**
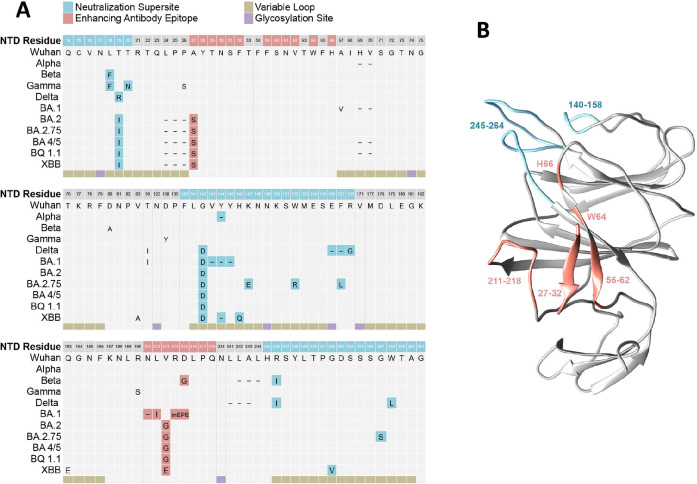
**Mutations in the S1 NTD of SARS-CoV-2 variants of concern.** (A) Sequence alignment of the N-terminal domain (NTD) of the SARS-CoV-2 spike protein from ancestral (Wuhan) and variant strains. Structural features include 5 hypervariable loops (beige) and glycosylation sites (purple), mutations mapping to regions within the convergent neutralization supersite (blue), and residues implicated in binding infection-enhancing mAbs (red). (B) Model of the 3-dimensional structure of SARS-CoV-2 NTD (PDB ID 6VSB) highlighting residues involved in antibody-mediated neutralization (blue) or infection-enhancement (red). Visualization was performed using UCSF Chimera, and unresolved loops were modeled using the Modeller web server [47, 48].

Our results also demonstrate loss of NTD antibody binding and functional activity to SARS-CoV-2 Omicron BA.1 and BA.2 variants. Comparison of NTD mutations in BA.1 relative to ancestral SARS-CoV-2 demonstrates multiple changes in positions 211-214, which encompass residues reported to impact binding of infection-enhancing mAbs (Figure 6) [19, 20]. Mutations in this region are also present in the NTD of Beta (D215G) and Omicron BA.2 (V213G), of which the latter change is maintained in all recent Omicron lineages, including BA2.75, BA 4/5, BQ1.1, and XBB (Figure 6A). Codons from 213-216 are also sites of recurring insertion elements among different SARS-CoV-2 lineages [46]; however, it is not known whether these recurrent insertions arise in response to immune selection or reflect compensatory changes that favor increased virus infectivity [46]. Additional mutations in the NTD spanning positions 24-27 also persist in all Omicron lineages, and these are directly adjacent to residues 27-32 implicated in the binding of infection-enhancing mAbs [19]. Taken together, mutations in the NTD constituting the neutralization supersite (residues 14–20, 140–158, and 245–264) and those implicated in binding infection-enhancing mAbs [19, 20] constitute most of the persisting changes in the NTD sequence of SARS-CoV-2 variants (Figure 6A) and impact discrete domains in the NTD protein (Figure 6B). While counterintuitive, our results support the thesis raised by others [20] that certain NTD-binding mAbs, while mediating enhanced infection with SARS-CoV-2 *in vitro*, may have protective FcγR-effector functions *in vivo*, with the potential to drive immune selection and NTD sequence variability at this site.

Importantly, our results demonstrate for the first time that NTD-binding, infection-enhancing antibodies are present as major serum clonotypes that can last in circulation for months during COVID-19 convalescence. Prior reports have described infection-enhancing mAbs derived from SARS-CoV-2 S-reactive plasmablasts or memory B-cell populations [19, 20]; ours is the first to identify NTD-binding, infection-enhancing mAbs isolated directly from serum (ie, functionally secreted antibody). While these serum-derived NTD-binding antibodies enhance SARS-CoV-2 PV infection *in vitro*, they also mediate activation of FcγRIIIa in response to binding SARS-CoV-2 S proteins, suggesting the potential for broader functionality that may include protective ADCC responses *in vivo*. FcγRIIIa activation by these antibodies was almost completely abrogated to BA.1 and BA.2 S proteins, again suggesting that mutations in the regions of the NTD recognized by these antibodies may contribute to a loss of functionality.

## CONCLUSIONS

Overall, our results suggest that enhancement of SARS-CoV-2 infection in the context of NTD-binding mAbs may be a unique phenomenon observed under certain *in vitro* conditions. This attribute, along with reports of their characteristic downward angle of binding to the SARS-CoV-2 S protein [19, 20] may help distinguish these mAbs from other NTD-binding antibodies, including those with neutralizing activity. In a broader context, our data support the concept that these antibodies arise during natural infection and represent a significant proportion of the memory B-cell antibody repertoire during convalescence. Evidence from this study demonstrates these NTD-binding, infection-enhancing mAbs can also be present as major secreted clonotypes in serum. They are able to activate cells through FcγRIIIa, which raises the possibility they may mediate protective FcR-effector functions *in vivo*. Further studies are needed to determine whether these antibodies contribute to immune protection, and whether they are induced by natural infection with SARS-CoV-2 variants or by immunization with next generation multivalent vaccines.

## References

[1] Lv Z, Deng YQ, Ye Q, Cao L, Sun CY, Fan C, Huang W, Sun S, Sun Y, Zhu L, Chen Q, Wang N, Nie J, Cui Z, Zhu D, Shaw N, Li XF, Li Q, Xie L, Wang Y, Rao Z, Qin CF, Wang X. Structural basis for neutralization of SARS-CoV-2 and SARS-CoV by a potent therapeutic antibody. *Science*. 2020;369(6510):1505–9. doi: 10.1126/science.abc5881. PubMed PMID: 32703908; PMCID: PMC7402622. 32703908 PMC7402622

[2] Salazar E, Kuchipudi SV, Christensen PA, Eagar T, Yi X, Zhao P, Jin Z, Long SW, Olsen RJ, Chen J, Castillo B, Leveque C, Towers D, Lavinder J, Gollihar J, Cardona J, Ippolito G, Nissly R, Bird I, Greenawalt D, Rossi RM, Gontu A, Srinivasan S, Poojary I, Cattadori IM, Hudson PJ, Josleyn NM, Prugar L, Huie K, Herbert A, Bernard DW, Dye JM, Kapur V, Musser JM. Convalescent plasma anti-SARS-CoV-2 spike protein ectodomain and receptor-binding domain IgG correlate with virus neutralization. *J Clin Invest*. 2020;130(12):6728–38. doi: 10.1172/JCI141206. PubMed PMID: 32910806; PMCID: PMC7685744. 32910806 PMC7685744

[3] Stamatatos L, Czartoski J, Wan YH, Homad LJ, Rubin V, Glantz H, Neradilek M, Seydoux E, Jennewein MF, MacCamy AJ, Feng J, Mize G, De Rosa SC, Finzi A, Lemos MP, Cohen KW, Moodie Z, McElrath MJ, McGuire AT. mRNA vaccination boosts cross-variant neutralizing antibodies elicited by SARS-CoV-2 infection. *Science*. 2021;372(6549):1413–8. doi: 10.1126/science.abg9175. PubMed PMID: 33766944; PMCID: PMC8139425. 33766944 PMC8139425

[4] Wec AZ, Wrapp D, Herbert AS, Maurer DP, Haslwanter D, Sakharkar M, Jangra RK, Dieterle ME, Lilov A, Huang D, Tse LV, Johnson NV, Hsieh CL, Wang N, Nett JH, Champney E, Burnina I, Brown M, Lin S, Sinclair M, Johnson C, Pudi S, Bortz R, 3rd, Wirchnianski AS, Laudermilch E, Florez C, Fels JM, O'Brien CM, Graham BS, Nemazee D, Burton DR, Baric RS, Voss JE, Chandran K, Dye JM, McLellan JS, Walker LM. Broad neutralization of SARS-related viruses by human monoclonal antibodies. *Science*. 2020;369(6504):731–6. doi: 10.1126/science.abc7424. PubMed PMID: 32540900; PMCID: PMC7299279. 32540900 PMC7299279

[5] Chen RE, Zhang X, Case JB, Winkler ES, Liu Y, VanBlargan LA, Liu J, Errico JM, Xie X, Suryadevara N, Gilchuk P, Zost SJ, Tahan S, Droit L, Turner JS, Kim W, Schmitz AJ, Thapa M, Wang D, Boon ACM, Presti RM, O'Halloran JA, Kim AHJ, Deepak P, Pinto D, Fremont DH, Crowe JE, Jr., Corti D, Virgin HW, Ellebedy AH, Shi PY, Diamond MS. Resistance of SARS-CoV-2 variants to neutralization by monoclonal and serum-derived polyclonal antibodies. *Nat Med*. 2021;27(4):717–26. doi: 10.1038/s41591-021-01294-w. PubMed PMID: 33664494; PMCID: PMC8058618. 33664494 PMC8058618

[6] Garcia-Beltran WF, Lam EC, St Denis K, Nitido AD, Garcia ZH, Hauser BM, Feldman J, Pavlovic MN, Gregory DJ, Poznansky MC, Sigal A, Schmidt AG, Iafrate AJ, Naranbhai V, Balazs AB. Multiple SARS-CoV-2 variants escape neutralization by vaccine-induced humoral immunity. *Cell*. 2021;184(9):2372–83 e9. doi: 10.1016/j.cell.2021.03.013. PubMed PMID: 33743213; PMCID: PMC7953441. 33743213 PMC7953441

[7] Liu Z, VanBlargan LA, Bloyet LM, Rothlauf PW, Chen RE, Stumpf S, Zhao H, Errico JM, Theel ES, Liebeskind MJ, Alford B, Buchser WJ, Ellebedy AH, Fremont DH, Diamond MS, Whelan SPJ. Identification of SARS-CoV-2 spike mutations that attenuate monoclonal and serum antibody neutralization. *Cell Host Microbe*. 2021;29(3):477–88 e4. doi: 10.1016/j.chom.2021.01.014. PubMed PMID: 33535027; PMCID: PMC7839837. 33535027 PMC7839837

[8] Harvey WT, Carabelli AM, Jackson B, Gupta RK, Thomson EC, Harrison EM, Ludden C, Reeve R, Rambaut A, Consortium C-GU, Peacock SJ, Robertson DL. SARS-CoV-2 variants, spike mutations and immune escape. *Nat Rev Microbiol*. 2021;19(7):409–24. doi: 10.1038/s41579-021-00573-0. PubMed PMID: 34075212; PMCID: PMC8167834. 34075212 PMC8167834

[9] Cantoni D, Murray MJ, Kalemera MD, Dicken SJ, Stejskal L, Brown G, Lytras S, Coey JD, McKenna J, Bridgett S, Simpson D, Fairley D, Thorne LG, Reuschl AK, Forrest C, Ganeshalingham M, Muir L, Palor M, Jarvis L, Willett B, Power UF, McCoy LE, Jolly C, Towers GJ, Doores KJ, Robertson DL, Shepherd AJ, Reeves MB, Bamford CGG, Grove J. Evolutionary remodelling of N-terminal domain loops fine-tunes SARS-CoV-2 spike. *EMBO Rep*. 2022;23(10):e54322. doi: 10.15252/embr.202154322. PubMed PMID: 35999696; PMCID: PMC9535765. 35999696 PMC9535765

[10] Gong Y, Qin S, Dai L, Tian Z. The glycosylation in SARS-CoV-2 and its receptor ACE2. *Signal Transduct Target Ther*. 2021;6(1):396. doi: 10.1038/s41392-021-00809-8. PubMed PMID: 34782609; PMCID: PMC8591162. 34782609 PMC8591162

[11] Seyran M, Takayama K, Uversky VN, Lundstrom K, Palu G, Sherchan SP, Attrish D, Rezaei N, Aljabali AAA, Ghosh S, Pizzol D, Chauhan G, Adadi P, Mohamed Abd El-Aziz T, Soares AG, Kandimalla R, Tambuwala M, Hassan SS, Azad GK, Pal Choudhury P, Baetas-da-Cruz W, Serrano-Aroca A, Brufsky AM, Uhal BD. The structural basis of accelerated host cell entry by SARS-CoV-2dagger. *FEBS J*. 2021;288(17):5010–20. doi: 10.1111/febs.15651. PubMed PMID: 33264497; PMCID: PMC7753708. 33264497 PMC7753708

[12] Soh WTL, Y.; Nakayama, E. E.; Ono, C.; Torii, S.; Nakagami, H.; Matsuura, Y.; Shioda, T.; Arase, H. The N-terminal domain of spike glycoprotein mediates SARS-CoV-2 infection by associating with L-SIGN and DC-SIGN. *bioRxiv*. 2020. doi: 10.1101/2020.11.05.369264.

[13] Cerutti G, Guo Y, Zhou T, Gorman J, Lee M, Rapp M, Reddem ER, Yu J, Bahna F, Bimela J, Huang Y, Katsamba PS, Liu L, Nair MS, Rawi R, Olia AS, Wang P, Zhang B, Chuang GY, Ho DD, Sheng Z, Kwong PD, Shapiro L. Potent SARS-CoV-2 neutralizing antibodies directed against spike N-terminal domain target a single supersite. *Cell Host Microbe*. 2021;29(5):819–33 e7. doi: 10.1016/j.chom.2021.03.005. PubMed PMID: 33789084; PMCID: PMC7953435. 33789084 PMC7953435

[14] Chi X, Yan R, Zhang J, Zhang G, Zhang Y, Hao M, Zhang Z, Fan P, Dong Y, Yang Y, Chen Z, Guo Y, Zhang J, Li Y, Song X, Chen Y, Xia L, Fu L, Hou L, Xu J, Yu C, Li J, Zhou Q, Chen W. A neutralizing human antibody binds to the N-terminal domain of the Spike protein of SARS-CoV-2. *Science*. 2020;369(6504):650–5. doi: 10.1126/science.abc6952. PubMed PMID: 32571838; PMCID: PMC7319273. 32571838 PMC7319273

[15] McCallum M, De Marco A, Lempp FA, Tortorici MA, Pinto D, Walls AC, Beltramello M, Chen A, Liu Z, Zatta F, Zepeda S, di Iulio J, Bowen JE, Montiel-Ruiz M, Zhou J, Rosen LE, Bianchi S, Guarino B, Fregni CS, Abdelnabi R, Foo SC, Rothlauf PW, Bloyet LM, Benigni F, Cameroni E, Neyts J, Riva A, Snell G, Telenti A, Whelan SPJ, Virgin HW, Corti D, Pizzuto MS, Veesler D. N-terminal domain antigenic mapping reveals a site of vulnerability for SARS-CoV-2. *Cell*. 2021;184(9):2332–47 e16. doi: 10.1016/j.cell.2021.03.028. PubMed PMID: 33761326; PMCID: PMC7962585. 33761326 PMC7962585

[16] Suryadevara N, Shrihari S, Gilchuk P, VanBlargan LA, Binshtein E, Zost SJ, Nargi RS, Sutton RE, Winkler ES, Chen EC, Fouch ME, Davidson E, Doranz BJ, Chen RE, Shi PY, Carnahan RH, Thackray LB, Diamond MS, Crowe JE, Jr. Neutralizing and protective human monoclonal antibodies recognizing the N-terminal domain of the SARS-CoV-2 spike protein. *Cell*. 2021;184(9):2316–31 e15. doi: 10.1016/j.cell.2021.03.029. PubMed PMID: 33773105; PMCID: PMC7962591. 33773105 PMC7962591

[17] Voss WN, Hou YJ, Johnson NV, Delidakis G, Kim JE, Javanmardi K, Horton AP, Bartzoka F, Paresi CJ, Tanno Y, Chou CW, Abbasi SA, Pickens W, George K, Boutz DR, Towers DM, McDaniel JR, Billick D, Goike J, Rowe L, Batra D, Pohl J, Lee J, Gangappa S, Sambhara S, Gadush M, Wang N, Person MD, Iverson BL, Gollihar JD, Dye JM, Herbert AS, Finkelstein IJ, Baric RS, McLellan JS, Georgiou G, Lavinder JJ, Ippolito GC. Prevalent, protective, and convergent IgG recognition of SARS-CoV-2 non-RBD spike epitopes. *Science*. 2021;372(6546):1108–12. doi: 10.1126/science.abg5268. PubMed PMID: 33947773; PMCID: PMC8224265. 33947773 PMC8224265

[18] Wang Z, Muecksch F, Cho A, Gaebler C, Hoffmann HH, Ramos V, Zong S, Cipolla M, Johnson B, Schmidt F, DaSilva J, Bednarski E, Ben Tanfous T, Raspe R, Yao K, Lee YE, Chen T, Turroja M, Milard KG, Dizon J, Kaczynska A, Gazumyan A, Oliveira TY, Rice CM, Caskey M, Bieniasz PD, Hatziioannou T, Barnes CO, Nussenzweig MC. Analysis of memory B cells identifies conserved neutralizing epitopes on the N-terminal domain of variant SARS-Cov-2 spike proteins. *Immunity*. 2022;55(6):998–1012 e8. doi: 10.1016/j.immuni.2022.04.003. PubMed PMID: 35447092; PMCID: PMC8986478. 35447092 PMC8986478

[19] Li D, Edwards RJ, Manne K, Martinez DR, Schafer A, Alam SM, Wiehe K, Lu X, Parks R, Sutherland LL, Oguin TH, 3rd, McDanal C, Perez LG, Mansouri K, Gobeil SMC, Janowska K, Stalls V, Kopp M, Cai F, Lee E, Foulger A, Hernandez GE, Sanzone A, Tilahun K, Jiang C, Tse LV, Bock KW, Minai M, Nagata BM, Cronin K, Gee-Lai V, Deyton M, Barr M, Von Holle T, Macintyre AN, Stover E, Feldman J, Hauser BM, Caradonna TM, Scobey TD, Rountree W, Wang Y, Moody MA, Cain DW, DeMarco CT, Denny TN, Woods CW, Petzold EW, Schmidt AG, Teng IT, Zhou T, Kwong PD, Mascola JR, Graham BS, Moore IN, Seder R, Andersen H, Lewis MG, Montefiori DC, Sempowski GD, Baric RS, Acharya P, Haynes BF, Saunders KO. In vitro and in vivo functions of SARS-CoV-2 infection-enhancing and neutralizing antibodies. *Cell*. 2021;184(16):4203–19 e32. doi: 10.1016/j.cell.2021.06.021. PubMed PMID: 34242577; PMCID: PMC8232969. 34242577 PMC8232969

[20] Liu Y, Soh WT, Kishikawa JI, Hirose M, Nakayama EE, Li S, Sasai M, Suzuki T, Tada A, Arakawa A, Matsuoka S, Akamatsu K, Matsuda M, Ono C, Torii S, Kishida K, Jin H, Nakai W, Arase N, Nakagawa A, Matsumoto M, Nakazaki Y, Shindo Y, Kohyama M, Tomii K, Ohmura K, Ohshima S, Okamoto T, Yamamoto M, Nakagami H, Matsuura Y, Nakagawa A, Kato T, Okada M, Standley DM, Shioda T, Arase H. An infectivity-enhancing site on the SARS-CoV-2 spike protein targeted by antibodies. *Cell*. 2021;184(13):3452–66 e18. doi: 10.1016/j.cell.2021.05.032. PubMed PMID: 34139176; PMCID: PMC8142859. 34139176 PMC8142859

[21] Beaudoin-Bussieres G, Chen Y, Ullah I, Prevost J, Tolbert WD, Symmes K, Ding S, Benlarbi M, Gong SY, Tauzin A, Gasser R, Chatterjee D, Vezina D, Goyette G, Richard J, Zhou F, Stamatatos L, McGuire AT, Charest H, Roger M, Pozharski E, Kumar P, Mothes W, Uchil PD, Pazgier M, Finzi A. A Fc-enhanced NTD-binding non-neutralizing antibody delays virus spread and synergizes with a nAb to protect mice from lethal SARS-CoV-2 infection. *Cell Rep*. 2022;38(7):110368. doi: 10.1016/j.celrep.2022.110368. PubMed PMID: 35123652; PMCID: PMC8786652. 35123652 PMC8786652

[22] Sakharkar M, Rappazzo CG, Wieland-Alter WF, Hsieh CL, Wrapp D, Esterman ES, Kaku CI, Wec AZ, Geoghegan JC, McLellan JS, Connor RI, Wright PF, Walker LM. Prolonged evolution of the human B cell response to SARS-CoV-2 infection. *Sci Immunol*. 2021;6(56). doi: 10.1126/sciimmunol.abg6916. PubMed PMID: 33622975; PMCID: PMC8128290. PMC8128290 33622975

[23] Wright PF, Prevost-Reilly AC, Natarajan H, Brickley EB, Connor RI, Wieland-Alter WF, Miele AS, Weiner JA, Nerenz RD, Ackerman ME. Longitudinal Systemic and Mucosal Immune Responses to SARS-CoV-2 Infection. *J Infect Dis*. 2022;226(7):1204–14. doi: 10.1093/infdis/jiac065. PubMed PMID: 35188974; PMCID: PMC8903457. 35188974 PMC8903457

[24] Butler SE, Crowley AR, Natarajan H, Xu S, Weiner JA, Bobak CA, Mattox DE, Lee J, Wieland-Alter W, Connor RI, Wright PF, Ackerman ME. Distinct Features and Functions of Systemic and Mucosal Humoral Immunity Among SARS-CoV-2 Convalescent Individuals. *Front Immunol*. 2020;11:618685. doi: 10.3389/fimmu.2020.618685. PubMed PMID: 33584712; PMCID: PMC7876222. 33584712 PMC7876222

[25] Natarajan H, Crowley AR, Butler SE, Xu S, Weiner JA, Bloch EM, Littlefield K, Wieland-Alter W, Connor RI, Wright PF, Benner SE, Bonny TS, Laeyendecker O, Sullivan D, Shoham S, Quinn TC, Larman HB, Casadevall A, Pekosz A, Redd AD, Tobian AAR, Ackerman ME. Markers of Polyfunctional SARS-CoV-2 Antibodies in Convalescent Plasma. *mBio*. 2021;12(2). doi: 10.1128/mBio.00765-21. PubMed PMID: 33879585; PMCID: PMC8092262. PMC8092262 33879585

[26] Curtis NC, Shin S, Hederman AP, Connor RI, Wieland-Alter WF, Ionov S, Boylston J, Rose J, Sakharkar M, Dorman DB, Dessaint JA, Gwilt LL, Crowley AR, Feldman J, Hauser BM, Schmidt AG, Ashare A, Walker LM, Wright PF, Ackerman ME, Lee J. Characterization of SARS-CoV-2 Convalescent Patients' Serological Repertoire Reveals High Prevalence of Iso-RBD Antibodies. *bioRxiv*. 2023. doi: 10.1101/2023.09.08.556349. PubMed PMID: 37745524; PMCID: PMC10515772.

[27] Letko M, Marzi A, Munster V. Functional assessment of cell entry and receptor usage for SARS-CoV-2 and other lineage B betacoronaviruses. *Nat Microbiol*. 2020;5(4):562–9. doi: 10.1038/s41564-020-0688-y. PubMed PMID: 32094589; PMCID: PMC7095430. 32094589 PMC7095430

[28] Cho H, Gonzales-Wartz KK, Huang D, Yuan M, Peterson M, Liang J, Beutler N, Torres JL, Cong Y, Postnikova E, Bangaru S, Talana CA, Shi W, Yang ES, Zhang Y, Leung K, Wang L, Peng L, Skinner J, Li S, Wu NC, Liu H, Dacon C, Moyer T, Cohen M, Zhao M, Lee FE, Weinberg RS, Douagi I, Gross R, Schmaljohn C, Pegu A, Mascola JR, Holbrook M, Nemazee D, Rogers TF, Ward AB, Wilson IA, Crompton PD, Tan J. Bispecific antibodies targeting distinct regions of the spike protein potently neutralize SARS-CoV-2 variants of concern. *Sci Transl Med*. 2021;13(616):eabj5413. doi: 10.1126/scitranslmed.abj5413. PubMed PMID: 34519517; PMCID: PMC8651051. 34519517 PMC8651051

[29] Garcia-Beltran WF, St Denis KJ, Hoelzemer A, Lam EC, Nitido AD, Sheehan ML, Berrios C, Ofoman O, Chang CC, Hauser BM, Feldman J, Roederer AL, Gregory DJ, Poznansky MC, Schmidt AG, Iafrate AJ, Naranbhai V, Balazs AB. mRNA-based COVID-19 vaccine boosters induce neutralizing immunity against SARS-CoV-2 Omicron variant. *Cell*. 2022;185(3):457–66 e4. doi: 10.1016/j.cell.2021.12.033. PubMed PMID: 34995482; PMCID: PMC8733787. 34995482 PMC8733787

[30] Dacon C, Tucker C, Peng L, Lee CD, Lin TH, Yuan M, Cong Y, Wang L, Purser L, Williams JK, Pyo CW, Kosik I, Hu Z, Zhao M, Mohan D, Cooper AJR, Peterson M, Skinner J, Dixit S, Kollins E, Huzella L, Perry D, Byrum R, Lembirik S, Drawbaugh D, Eaton B, Zhang Y, Yang ES, Chen M, Leung K, Weinberg RS, Pegu A, Geraghty DE, Davidson E, Douagi I, Moir S, Yewdell JW, Schmaljohn C, Crompton PD, Holbrook MR, Nemazee D, Mascola JR, Wilson IA, Tan J. Broadly neutralizing antibodies target the coronavirus fusion peptide. *Science*. 2022;377(6607):728–35. doi: 10.1126/science.abq3773. PubMed PMID: 35857439; PMCID: PMC9348754. 35857439 PMC9348754

[31] Yahi N, Chahinian H, Fantini J. Infection-enhancing anti-SARS-CoV-2 antibodies recognize both the original Wuhan/D614G strain and Delta variants. A potential risk for mass vaccination? *J Infect*. 2021;83(5):607–35. doi: 10.1016/j.jinf.2021.08.010. PubMed PMID: 34384810; PMCID: PMC8351274. PMC8351274 34384810

[32] Aguiar JA, Tremblay BJ, Mansfield MJ, Woody O, Lobb B, Banerjee A, Chandiramohan A, Tiessen N, Cao Q, Dvorkin-Gheva A, Revill S, Miller MS, Carlsten C, Organ L, Joseph C, John A, Hanson P, Austin RC, McManus BM, Jenkins G, Mossman K, Ask K, Doxey AC, Hirota JA. Gene expression and in situ protein profiling of candidate SARS-CoV-2 receptors in human airway epithelial cells and lung tissue. *Eur Respir J*. 2020;56(3). doi: 10.1183/13993003.01123-2020. PubMed PMID: 32675206; PMCID: PMC7366180. PMC7366180 32675206

[33] Hou YJ, Okuda K, Edwards CE, Martinez DR, Asakura T, Dinnon KH, 3rd, Kato T, Lee RE, Yount BL, Mascenik TM, Chen G, Olivier KN, Ghio A, Tse LV, Leist SR, Gralinski LE, Schafer A, Dang H, Gilmore R, Nakano S, Sun L, Fulcher ML, Livraghi-Butrico A, Nicely NI, Cameron M, Cameron C, Kelvin DJ, de Silva A, Margolis DM, Markmann A, Bartelt L, Zumwalt R, Martinez FJ, Salvatore SP, Borczuk A, Tata PR, Sontake V, Kimple A, Jaspers I, O'Neal WK, Randell SH, Boucher RC, Baric RS. SARS-CoV-2 Reverse Genetics Reveals a Variable Infection Gradient in the Respiratory Tract. *Cell*. 2020;182(2):429–46 e14. doi: 10.1016/j.cell.2020.05.042. PubMed PMID: 32526206; PMCID: PMC7250779. 32526206 PMC7250779

[34] Li MY, Li L, Zhang Y, Wang XS. Expression of the SARS-CoV-2 cell receptor gene ACE2 in a wide variety of human tissues. *Infect Dis Poverty*. 2020;9(1):45. doi: 10.1186/s40249-020-00662-x. PubMed PMID: 32345362; PMCID: PMC7186534. 32345362 PMC7186534

[35] Wang Y, Wang Y, Luo W, Huang L, Xiao J, Li F, Qin S, Song X, Wu Y, Zeng Q, Jin F, Wang Y. A comprehensive investigation of the mRNA and protein level of ACE2, the putative receptor of SARS-CoV-2, in human tissues and blood cells. *Int J Med Sci*. 2020;17(11):1522–31. doi: 10.7150/ijms.46695. PubMed PMID: 32669955; PMCID: PMC7359402. 32669955 PMC7359402

[36] ter Meulen J, van den Brink EN, Poon LL, Marissen WE, Leung CS, Cox F, Cheung CY, Bakker AQ, Bogaards JA, van Deventer E, Preiser W, Doerr HW, Chow VT, de Kruif J, Peiris JS, Goudsmit J. Human monoclonal antibody combination against SARS coronavirus: synergy and coverage of escape mutants. *PLoS Med*. 2006;3(7):e237. doi: 10.1371/journal.pmed.0030237. PubMed PMID: 16796401; PMCID: PMC1483912. 16796401 PMC1483912

[37] Wang Z, Muecksch F, Schaefer-Babajew D, Finkin S, Viant C, Gaebler C, Hoffmann HH, Barnes CO, Cipolla M, Ramos V, Oliveira TY, Cho A, Schmidt F, Da Silva J, Bednarski E, Aguado L, Yee J, Daga M, Turroja M, Millard KG, Jankovic M, Gazumyan A, Zhao Z, Rice CM, Bieniasz PD, Caskey M, Hatziioannou T, Nussenzweig MC. Naturally enhanced neutralizing breadth against SARS-CoV-2 one year after infection. *Nature*. 2021;595(7867):426–31. doi: 10.1038/s41586-021-03696-9. PubMed PMID: 34126625; PMCID: PMC8277577. 34126625 PMC8277577

[38] Yuan M, Wu NC, Zhu X, Lee CD, So RTY, Lv H, Mok CKP, Wilson IA. A highly conserved cryptic epitope in the receptor binding domains of SARS-CoV-2 and SARS-CoV. *Science*. 2020;368(6491):630–3. doi: 10.1126/science.abb7269. PubMed PMID: 32245784; PMCID: PMC7164391. 32245784 PMC7164391

[39] Chu H, Chan JF, Yuen TT, Shuai H, Yuan S, Wang Y, Hu B, Yip CC, Tsang JO, Huang X, Chai Y, Yang D, Hou Y, Chik KK, Zhang X, Fung AY, Tsoi HW, Cai JP, Chan WM, Ip JD, Chu AW, Zhou J, Lung DC, Kok KH, To KK, Tsang OT, Chan KH, Yuen KY. Comparative tropism, replication kinetics, and cell damage profiling of SARS-CoV-2 and SARS-CoV with implications for clinical manifestations, transmissibility, and laboratory studies of COVID-19: an observational study. *Lancet Microbe*. 2020;1(1):e14–e23. doi: 10.1016/S2666-5247(20)30004-5. PubMed PMID: 32835326; PMCID: PMC7173822. 32835326 PMC7173822

[40] Feng XL, Yu D, Zhang M, Li X, Zou QC, Ma W, Han JB, Xu L, Yang C, Qu W, Deng ZH, Long J, Long Y, Li M, Yao YG, Dong XQ, Zeng J, Li MH. Characteristics of replication and pathogenicity of SARS-CoV-2 Alpha and Delta isolates. *Virol Sin*. 2022;37(6):804–12. doi: 10.1016/j.virs.2022.09.007. PubMed PMID: 36167254; PMCID: PMC9507998. 36167254 PMC9507998

[41] Jureka AS, Basler CF. Propagation and Quantification of SARS-CoV-2. *Methods Mol Biol*. 2022;2452:111–29. doi: 10.1007/978-1-0716-2111-0_8. PubMed PMID: 35554904. 35554904

[42] Mautner L, Hoyos M, Dangel A, Berger C, Ehrhardt A, Baiker A. Replication kinetics and infectivity of SARS-CoV-2 variants of concern in common cell culture models. *Virol J*. 2022;19(1):76. doi: 10.1186/s12985-022-01802-5. PubMed PMID: 35473640; PMCID: PMC9038516. 35473640 PMC9038516

[43] Rajah MM, Hubert M, Bishop E, Saunders N, Robinot R, Grzelak L, Planas D, Dufloo J, Gellenoncourt S, Bongers A, Zivaljic M, Planchais C, Guivel-Benhassine F, Porrot F, Mouquet H, Chakrabarti LA, Buchrieser J, Schwartz O. SARS-CoV-2 Alpha, Beta, and Delta variants display enhanced Spike-mediated syncytia formation. *EMBO J*. 2021;40(24):e108944. doi: 10.15252/embj.2021108944. PubMed PMID: 34601723; PMCID: PMC8646911. 34601723 PMC8646911

[44] Ren X, Glende J, Al-Falah M, de Vries V, Schwegmann-Wessels C, Qu X, Tan L, Tschernig T, Deng H, Naim HY, Herrler G. Analysis of ACE2 in polarized epithelial cells: surface expression and function as receptor for severe acute respiratory syndrome-associated coronavirus. *J Gen Virol*. 2006;87(Pt 6):1691–5. doi: 10.1099/vir.0.81749-0. PubMed PMID: 16690935. 16690935

[45] Farrell AG, Dadonaite B, Greaney AJ, Eguia R, Loes AN, Franko NM, Logue J, Carreno JM, Abbad A, Chu HY, Matreyek KA, Bloom JD. Receptor-Binding Domain (RBD) Antibodies Contribute More to SARS-CoV-2 Neutralization When Target Cells Express High Levels of ACE2. *Viruses*. 2022;14(9). doi: 10.3390/v14092061. PubMed PMID: 36146867; PMCID: PMC9504593. PMC9504593 36146867

[46] Gerdol M, Dishnica K, Giorgetti A. Emergence of a recurrent insertion in the N-terminal domain of the SARS-CoV-2 spike glycoprotein. *Virus Res*. 2022;310:198674. doi: 10.1016/j.virusres.2022.198674. PubMed PMID: 35021068; PMCID: PMC8743576. 35021068 PMC8743576

[47] Pettersen EF, Goddard TD, Huang CC, Couch GS, Greenblatt DM, Meng EC, Ferrin TE. UCSF Chimera–a visualization system for exploratory research and analysis. *J Comput Chem*. 2004;25(13):1605–12. doi: 10.1002/jcc.20084. PubMed PMID: 15264254. 15264254

[48] Sali A. Comparative protein modeling by satisfaction of spatial restraints. *Mol Med Today*. 1995;1(6):270–7. doi: 10.1016/s1357-4310(95)91170-7. PubMed PMID: 9415161. 9415161

